# Estimating Leptospirosis Incidence Using Hospital-Based Surveillance and a Population-Based Health Care Utilization Survey in Tanzania

**DOI:** 10.1371/journal.pntd.0002589

**Published:** 2013-12-05

**Authors:** Holly M. Biggs, Julian T. Hertz, O. Michael Munishi, Renee L. Galloway, Florian Marks, Wilbrod Saganda, Venance P. Maro, John A. Crump

**Affiliations:** 1 Division of Infectious Diseases, Department of Medicine, Duke University Medical Center, Durham, North Carolina, United States of America; 2 Kilimanjaro Christian Medical Centre, Moshi, United Republic of Tanzania; 3 Centers for Disease Control and Prevention, Bacterial Special Pathogens Branch, Atlanta, Georgia, United States of America; 4 International Vaccine Institute, Seoul, South Korea; 5 Mawenzi Regional Hospital, Moshi, United Republic of Tanzania; 6 Kilimanjaro Christian Medical University College, Moshi, United Republic of Tanzania; 7 Duke Global Health Institute, Duke University, Durham, North Carolina, United States of America; Institute of Collective Health, Federal University of Bahia, Brazil

## Abstract

**Background:**

The incidence of leptospirosis, a neglected zoonotic disease, is uncertain in Tanzania and much of sub-Saharan Africa, resulting in scarce data on which to prioritize resources for public health interventions and disease control. In this study, we estimate the incidence of leptospirosis in two districts in the Kilimanjaro Region of Tanzania.

**Methodology/Principal Findings:**

We conducted a population-based household health care utilization survey in two districts in the Kilimanjaro Region of Tanzania and identified leptospirosis cases at two hospital-based fever sentinel surveillance sites in the Kilimanjaro Region. We used multipliers derived from the health care utilization survey and case numbers from hospital-based surveillance to calculate the incidence of leptospirosis. A total of 810 households were enrolled in the health care utilization survey and multipliers were derived based on responses to questions about health care seeking in the event of febrile illness. Of patients enrolled in fever surveillance over a 1 year period and residing in the 2 districts, 42 (7.14%) of 588 met the case definition for confirmed or probable leptospirosis. After applying multipliers to account for hospital selection, test sensitivity, and study enrollment, we estimated the overall incidence of leptospirosis ranges from 75–102 cases per 100,000 persons annually.

**Conclusions/Significance:**

We calculated a high incidence of leptospirosis in two districts in the Kilimanjaro Region of Tanzania, where leptospirosis incidence was previously unknown. Multiplier methods, such as used in this study, may be a feasible method of improving availability of incidence estimates for neglected diseases, such as leptospirosis, in resource constrained settings.

## Author Summary

Leptospirosis is a zoonotic infection that occurs worldwide and is caused by a spirochete, *Leptospira* spp. The incidence of leptospirosis is unknown in most of sub-Saharan Africa, including Tanzania. Incidence estimates are important in prioritizing resources for disease prevention and control. In this study, we calculated leptospirosis incidence in 2 districts in the Kilimanjaro Region of Tanzania using a multiplier method. We used responses from a population-based survey that asked where participants and their household members would seek health care in the event of fever along with the number of leptospirosis cases found at 2 hospitals under surveillance to calculate estimated incidence. We calculated a high incidence of leptospirosis in the study area that was previously unrecognized. This has important implications for prioritizing further research and consideration of public health control measures for leptospirosis in Tanzania.

## Introduction

Incidence estimates of infectious diseases are crucial for determining burden of disease and prioritizing resources for disease control. However, these estimates are often unavailable in resource constrained settings, resulting in scarce data on which to base recommendations for public health interventions. Active population-based surveillance, using door-to-door visits in the community, is an ideal method for measuring infectious disease incidence, but active surveillance is limited in many areas due to its requisite investment of time and resources. Previous studies have described methods for extrapolating data from hospital based surveillance and population-based surveys of health care seeking behavior to estimate disease incidence in a population [1]–[5]. This method has facilitated disease incidence estimates in populations in resource constrained settings where these data were previously unavailable.

The incidence of leptospirosis, a neglected, poverty-associated zoonosis found worldwide, is uncertain in sub-Saharan Africa [6]. Several studies in sub-Saharan African countries have shown that leptospirosis may comprise a substantial proportion of acute febrile illness [7]–[11]. However, population-based incidence estimates are lacking with the exception of studies from the Seychelles showing a high annual incidence of 60–101 cases per 100,000 persons [12], [13]. The lack of data is likely the consequence of limited access to laboratories with leptospirosis diagnostic capability, low clinician awareness of the disease, often nonspecific clinical features of leptospirosis, and lack of surveillance infrastructure. As a result, in sub-Saharan Africa public health measures for leptospirosis prevention and control have not been prioritized, and leptospirosis remains a neglected cause of febrile illness. In this study, we estimate leptospirosis incidence in two districts in the Kilimanjaro Region of Tanzania using data from hospital based surveillance and multipliers derived from a population-based household health care utilization survey.

## Methods

### Study site

**Figure 1:**
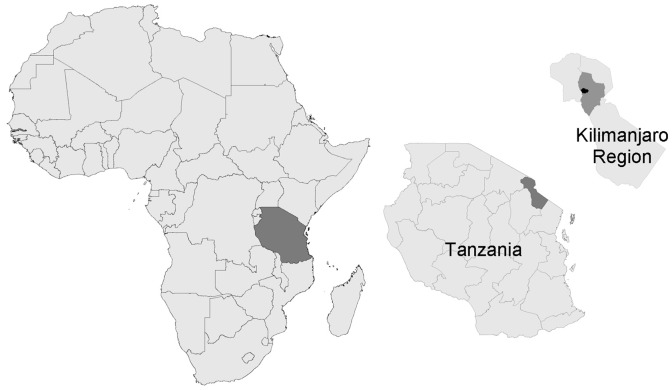
Africa, Tanzania and Kilimanjaro Region. Moshi Rural District shown in dark gray and Moshi Urban District in black in the Kilimanjaro Region inset.

This study was conducted in the Kilimanjaro Region in northern Tanzania. The household survey was done in 2 districts in the Kilimanjaro Region, Moshi Rural (population 401,369) and Moshi Urban (population 143,799) (Figure 1). Febrile illness surveillance was conducted at 2 hospitals in Moshi, Kilimanjaro Christian Medical Centre (KCMC) and Mawenzi Regional Hospital (MRH). These hospitals serve as major providers of care for residents of Moshi Urban and Moshi Rural. KCMC and MRH are located a diagonal distance of 3.5 km apart and a driving distance of approximately 5.5 km apart. KCMC is a 458 bed tertiary referral hospital that serves several regions in northern Tanzania, and MRH is a 300 bed regional hospital that serves the Kilimanjaro Region.

### Health care utilization survey

#### Household selection

Thirty (66.67%) of the 45 wards in Moshi Urban and Moshi Rural Districts were randomly selected in a population-weighted fashion, including 22 wards from Moshi Rural and 8 wards from Moshi Urban. Ward-level population data were taken from the 2002 Tanzanian National Census [14]. A starting point in each selected ward was chosen arbitrarily while touring the ward on foot by a member of the study team who was not previously familiar with the area. A direction was similarly chosen, and the first 27 households along that direction from the starting point were included in the survey.

#### Design and administration of the survey

The health care utilization survey was conducted from June 13, 2011 through July 22, 2011. After obtaining informed consent, members of the study team administered the survey to heads of the 27 selected households in each ward. The standardized survey included questions about demographics, socioeconomic status and health care seeking behavior. Two distinct sets of questions related to health care seeking behavior in the event of fever were asked. Heads of household were queried ‘what is the name of the health care facility with an inpatient ward where you/your family would go if you/your family had fever?’ Choices included all 8 hospitals serving Moshi Urban and Moshi Rural Districts, including MRH and KCMC, and ‘other’ with a free text field. For this question, one response per household was recorded without any age grouping. Heads of household were also asked about health care seeking behavior for household members in particular age groups (<1 year, 1 to <5 years, 5 to <15 years and ≥15 years). Respondents ranked their top 3 choices for response to ‘fever,’ ‘elevated body temperature <3 days,’ and ‘elevated body temperature ≥3 days.’ Options included ‘treatment at home,’ ‘traditional healer,’ ‘private clinic/health center,’ ‘government clinic/health center,’ ‘nothing,’ ‘MRH,’ and ‘other hospital’ with a pick list including the 7 other hospitals serving Moshi Urban and Moshi Rural Districts as well an option for ‘other’ unspecified hospital.

### Fever surveillance

As part of a comprehensive study of the etiology of febrile illness in northern Tanzania, adult and pediatric inpatients at KCMC and adult inpatients at MRH were prospectively enrolled from September 17, 2007 through August 31, 2008. Methods and results have been previously described [15]–[17]. Patients admitted to the adult medicine wards, aged ≥13 years, were eligible to participate if they had an oral temperature of ≥38.0°C and had been admitted for <24 hours. Pediatric inpatients, aged ≥2 months to <13 years, were eligible if they had a history of fever in the past 48 hours, an axillary temperature of ≥37.5°C or a rectal temperature of ≥38.0°C and had been admitted for <24 hours. Demographic information, including the participant's district and village of residence, was collected. Participants were asked whether they had been referred from another inpatient hospital. Acute serum was drawn and archived, and all participants were asked to return 4–6 weeks after enrollment to submit a convalescent serum sample. Acute and convalescent serum samples were sent to the United States Centers for Disease Control and Prevention (CDC) for serologic analysis for leptospirosis.

#### Laboratory methods

Leptospirosis laboratory diagnosis was made using the standard microscopic agglutination test (MAT) performed at the CDC using a panel of antigens from 20 different *Leptospira* serovars representing 17 serogroups [18]. Live leptospiral cell suspensions were incubated with serially diluted serum specimens. Resulting agglutination titers were read using darkfield microscopy. The reported titer was the highest dilution of serum that agglutinated at least 50% of the cells for each serovar tested [18].

The serogroups (serovars) included in the antigen panel were Australis (*L. interrogans* serovar Australis, *L. interrogans* serovar Bratislava), Autumnalis (*L. interrogans* serovar Autumnalis), Ballum (*L. borgpetersenii* serovar Ballum), Bataviae (*L. interrogans* serovar Bataviae), Canicola (*L. interrogans* serovar Canicola), Celledoni (*L. weilii* serovar Celledoni), Cynopteri (*L. kirschneri* serovar Cynopteri), Djasiman (*L. interrogans* serovar Djasiman), Grippotyphosa (*L. kirschneri* serovar Grippotyphosa), Hebdomadis (*L. santarosai* serovar Borincana), Icterohaemorrhagiae (*L. interrogans* serovar Mankarso, *L. interrogans* Icterohaemorrhagiae), Javanica (*L. borgpetersenii* serovar Javanica), Mini (*L. santarosai* serovar Georgia), Pomona (*L. interrogans* serovar Pomona), Pyrogenes (*L. interrogans* serovar Pyrogenes, *L. santarosai* serovar Alexi), Sejroe (*L. interrogans* serovar Wolffi), and Tarassovi (*L. borgpetersenii* serovar Tarassovi).

#### Study definitions

Confirmed leptospirosis was defined as a ≥4-fold rise in the agglutination titer between acute and convalescent serum samples [19]. Probable leptospirosis was defined as any single reciprocal MAT titer ≥800 among those not meeting the definition for confirmed leptospirosis [20]–[22].

### Incidence calculations

**Figure 2:**
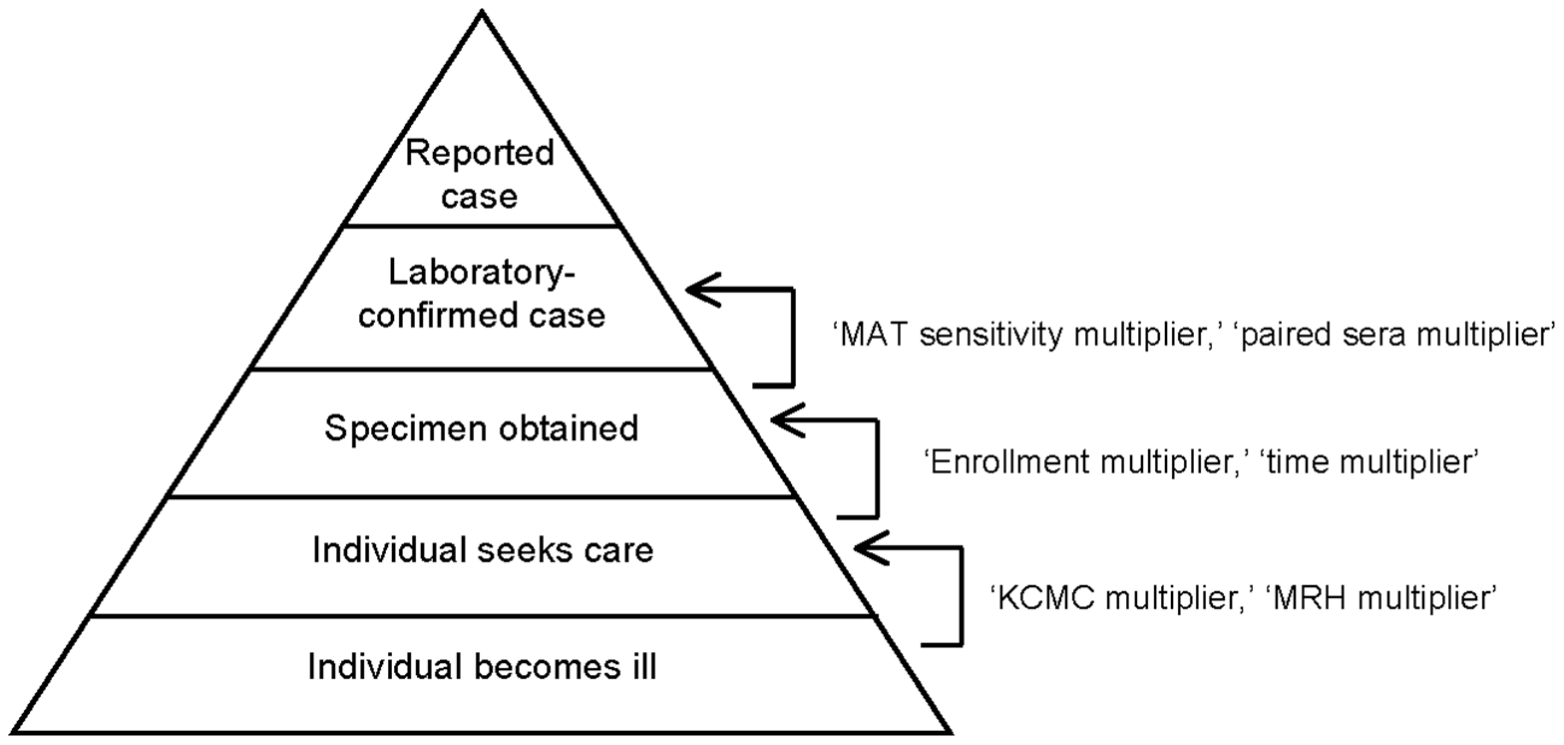
Surveillance pyramid. Multipliers were applied to account for incomplete assessments at various levels of the surveillance pyramid.

Incidence was estimated with the use of multipliers derived from the health care utilization survey and fever surveillance. Multipliers account for leptospirosis cases that were potentially missed in the stages of reporting (Figure 2) and are the multiplicative inverse of the relevant proportions.

We calculated the ‘KCMC multiplier’ and ‘MRH multiplier’ to account for health care seeking preferences and cases potentially missed due to selection of health care providers or options not under surveillance. In order to evaluate the sensitivity of our estimates, the ‘KCMC multiplier’ and ‘MRH multiplier’ were derived based on head of household responses to the 2 distinct survey questions: ‘What is the name of the health care facility with an inpatient ward where you/your family would go if you/your family had fever?’ and ‘What will you do if a member of this household in x age group has elevated body temperature for ≥3 days?.’ We selected the first and second choice responses to ‘elevated body temperature for ≥3 days’ as most representative of where patients sufficiently ill to warrant hospital admission would seek care. If the head of household's first choice for care in the event of ‘elevated body temperature for ≥3 days’ was KCMC and second choice was MRH, then we only counted the first choice. Since KCMC is a tertiary referral hospital, patients who would elect to present to KCMC first would be unlikely to subsequently be seen at MRH for the same illness. In addition, we calculated a ‘referral adjustment’ to adjust for patients transferred to KCMC from another inpatient hospital given that transfer may not reflect a patient's preference of health care facility.

We calculated an ‘enrollment multiplier’ to account for patients who were eligible but not enrolled in fever surveillance for any reason. We calculated a ‘time multiplier’ to account for fever surveillance enrollment 5 (71.43%) of 7 days of the week. We also calculated a ‘paired sera multiplier’ to account for patients in fever surveillance that did not have acute and convalescent serum samples tested, and therefore could not meet criteria for confirmed leptospirosis. This multiplier was applied to the incidence estimates involving only confirmed cases. We calculated a ‘MAT sensitivity multiplier’ to account for the sensitivity of the diagnostic test. MAT sensitivity on paired sera was estimated to approach 100%, while sensitivity on acute serum only was estimated at 48.7%, and sensitivity on convalescent serum only was estimated at 93.8% [23]. Case numbers were also adjusted to account for MAT specificity of approximately 97.3% [23]. Multiplier derivations based on study results are presented in detail in the results.

Incidence was calculated by age group as follows: age 0 to <5 years, age 5 to <15 years, and age ≥15 years. We used the 2002 Tanzania National Census, the most recent population data available for Tanzania, to determine the population of Moshi Urban and Moshi Rural for the specified age groups [14].

### Statistical analysis

Data were entered using the Cardiff Teleform system (Cardiff, Inc., Vista, CA, USA) into an Access database (Microsoft Corp, Redmond, WA). Incidence calculations were done using Microsoft Excel 2010 (Microsoft Corp. Redmond, WA) spreadsheets. Other analyses were performed using STATA, version 10.1 (STATACorp, College Station, TX) and Epi Info 7, version 7.1.2.0 (CDC, Atlanta, GA). Pearson's chi-square was used to compare the health care utilization study population with the census population. All p values are 2 sided and evaluated for statistical significance at the 0.05 significance level.

### Research ethics

This study was approved by the KCMC Research Ethics Committee, the Tanzania National Institutes for Medical Research National Research Ethics Coordinating Committee, and the Institutional Review Boards of Duke University Medical Center, the CDC, and the International Vaccine Institute. All study participants provided written informed consent.

## Results

### Health care utilization survey

In the health care utilization study, 810 households were enrolled; no selected household refused participation. Responses represented a total of 3919 household members. Table 1 shows the demographics of the health care utilization study population compared to the general census population of the two districts.

**Table 1:** Demographics of health care utilization study population compared to Tanzania 2002 Population and Housing Census, Moshi Urban and Moshi Rural Districts, combined.

	HCUS study population n = 3,119 no.(%)	Census population* n = 545,168 no.(%)	P value
Age group (years)			
0 to <5	225 (7.2)	68,680 (12.6)	<0.001
5 to <15	685 (22.0)	150,218 (27.6)	<0.001
≥15	2,209 (70.8)	326,270 (59.8)	<0.001
Sex			
Female	1,690/3066 (55.1)	282,252 (51.8)	<0.001
Male	1,376/3066 (44.9)	262,916 (48.2)	

* Ref 7.

All households had at least one member aged ≥15 years; 361 had ≥1 member aged 5 years to <15 years; 156 had ≥1 child aged 1 year to <5 years; and 42 had ≥1 infant <1 year. Responses for the <1 year and 1 to <5 years age groups were combined for analysis due to the small number of responses in the <1 year age group as well as to more closely match the age intervals of Tanzania census data. After combining these age groups, the <1 to 5 years age group had 198 responses; 16 households had members of both age groups for which responses to both questions were included. Aside from this exception, a response for a given age group was counted only once per household, regardless of the number of household members in that age group.

#### Multiplier derivation

The proportion of heads of household choosing MRH or KCMC for management of ‘elevated body temperature for ≥3 days’ and as the ‘health care facility with an inpatient ward where you/your family would go for fever?’ with resultant multipliers are shown in Table 2.

**Table 2:** Reported health care seeking behavior among household survey respondents and calculated multipliers, Moshi Urban and Moshi Rural Districts, Tanzania, 2011.

Age group (years)	No. responses	No. selecting health facility		KCMC multiplier‡	MRH multiplier§
		KCMC* no.(%)	MRH† no.(%)		
‘Elevated body temperature for 3 or more days’					
0 to <5	198	17(8.59)		11.65	
5 to <15	361	10(2.77)		36.10	
≥15	810	35(4.32)	290(35.80)	23.14	2.79
‘Health care facility with an inpatient ward where you/your family would go for fever?’					
	810	50(6.17)	313(38.64)	16.20	2.59

KCMC = Kilimanjaro Christian Medical Centre, MRH = Mawenzi Regional Hospital.

* Number of respondents who chose KCMC as their first or second choice for health care in response to respective questions.

† Number of respondents who chose MRH as their first or second choice for health care in response to respective questions.

‡ Inverse of proportion of respondents who select KCMC for care in response to respective questions.

§ Inverse of proportion of respondents who select MRH for care in response to respective questions.

### Fever surveillance

A total of 870 inpatients were enrolled at KCMC and MRH. Participant characteristics have been described elsewhere [15]–[17]. Residence in Moshi Urban or Moshi Rural Districts was reported by 588 (67.59%) participants. Of those residing in the study area, 315 (53.57%) of 588 had paired sera tested, 222 (37.76%) had only acute serum tested, and 28 (4.76%) had only convalescent serum tested. Of those with paired sera tested, 23 (7.30%) of 315 met the case definition for confirmed leptospirosis. Of those with ≥1 serum sample tested, and not classified as confirmed leptospirosis, 19 (3.51%) of 542 met the definition of probable leptospirosis. Case numbers by age group and enrollment site are shown in Table 3.

**Table 3:** Confirmed and probable leptospirosis cases, Moshi Urban and Moshi Rural Districts residents, Kilimanjaro Christian Medical Center (KCMC) and Mawenzi Regional Hospital (MRH), Tanzania 2007–2008.

Age group (years)	KCMC				MRH			
	Confirmed cases* no.	Probable cases no.	Probable cases, acute serum only† no.	Probable cases, paired serum* no.	Confirmed cases* no.	Probable cases no.	Probable cases, acute serum only† no.	Probable cases, paired serum* no.
0 to <5	7	1	0	1				
5 to <15	3	3	0	3				
≥15	1	2	1	1	12	13	10	3
Total	11	6	1	5	12	13	10	3

KCMC = Kilimanjaro Christian Medical Centre, MRH = Mawenzi Regional Hospital.

* Confirmed or probable cases based on testing of paired sera. For adjusted case calculations these cases were multiplied by a ‘MAT sensitivity multiplier’ of 1.00.

† Probable cases based on testing of acute serum only. For adjusted case calculations these cases were multiplied by a ‘MAT sensitivity multiplier’ of 2.05.

#### Multiplier derivation

Of patients screened for fever surveillance, 1,310 were eligible for enrollment and 870 (66.41%) were enrolled, resulting in an overall ‘enrollment multiplier’ of 1.51. Enrollment multipliers by age group are shown in Table 4.

**Table 4:** Leptospirosis incidence estimates, Moshi Urban and Moshi Rural Districts, Tanzania 2007–2008.

Age group (years)	KCMC		MRH		KCMC and MRH adjusted cases‡ no.	Time multiplier	Enrollment multiplier	Paired sera multiplier§	Annual estimated cases no.	Population	Annual incidence (per 100,000)#
	Crude cases no.	Adjusted cases* no.	Crude cases no.	Adjusted cases† no.							
‘Elevated body temperature for 3 or more days’; confirmed and probable leptospirosis cases											
0 to <5	8	61			61	1.4	1.41	NA	120	68,680	175
5 to <15	6	143			143	1.4	1.21	NA	242	150,218	161
≥15	3	71	25	96	84	1.4	1.65	NA	194	326,270	59
Overall									556	545,168	102
‘Elevated body temperature for 3 or more days’; confirmed leptospirosis cases											
0 to <5	7	54			54	1.4	1.41	1.86	198	68,680	288
5 to <15	3	71			71	1.4	1.21	1.86	224	150,218	149
≥15	1	18	12	32	25	1.4	1.65	1.86	107	326,270	33
Overall									529	545,168	97
‘Health care facility with an inpatient ward where you/your family would go for fever’; confirmed and probable leptospirosis cases¶											
	17	193				1.4	1.51	NA	408	545,168	75
‘Health care facility with an inpatient ward where you/your family would go for fever’; confirmed leptospirosis cases¶											
	11	118				1.4	1.51	1.86	464	545,168	85

KCMC = Kilimanjaro Christian Medical Centre, MRH = Mawenzi Regional Hospital, MAT = microscopic agglutination test.

* Cases adjusted by ‘MAT sensitivity multiplier,’ MAT specificity, ‘KCMC multiplier,’ and KCMC referral adjustment.

† Cases adjusted by ‘MAT sensitivity multiplier,’ MAT specificity and ‘MRH multiplier’.

‡ No. for the ≥15 years age group represents the mean of the KCMC adjusted case no. and MRH adjusted case no. No. for the 0 to <5 years and 5 to <15 years age groups equal the KCMC adjusted case no. since fever surveillance was not conducted in these groups at MRH.

§ ‘Paired sera multiplier’ applied to estimates using confirmed cases only.

¶ Only KCMC data used for this estimate since MRH data represents a limited age range.

# (Annual estimated cases/population) *100,000.

Of leptospirosis cases from Moshi Urban and Moshi Rural Districts, all had either paired sera or acute serum only tested; no case was defined based on convalescent serum alone. Therefore, MAT sensitivity multipliers of 1.00 and 2.05, respectively, were applied. Case numbers were also adjusted for MAT specificity by multiplying by 0.93. Of enrollees from Moshi Urban and Moshi Rural Districts, 316 (53.8%) of 588 had paired sera, resulting in a ‘paired sera multiplier’ of 1.86.

Of participants from Moshi Urban and Moshi Rural Districts admitted at KCMC, 100 (32.5%) of 308 infants and children and 20 (22.5%) of 89 adults reported transfer from another inpatient hospital. Crude case numbers for children and adults at KCMC were therefore adjusted by multiplying by 0.68 and 0.78, respectively.

### Incidence calculations

Incidence calculations using the 2 distinct fever-related questions as well as different leptospirosis case definitions are shown in Table 3. Leptospirosis incidence in Moshi Urban and Moshi Rural Districts by age group is estimated as follows: 0 to <5 years, 175–288 cases per 100,000 persons/year; 5 to <15 years, 149–161 cases per 100,000 persons/year; ≥15 years, 33–59 cases per 100,000 persons/year. Overall annual leptospirosis incidence in Moshi Urban and Moshi Rural is estimated at 97 to 102 cases per 100,000 persons based on responses to ‘elevated body temperature ≥3 days’ and 75 to 85 cases per 100,000 persons based on responses to ‘health care facility with an inpatient ward where you/your family would go if you/your family had fever?’. Our best estimate of overall incidence, including the most comprehensive use of data derived from the question about ‘elevated body temperature ≥3 days’ and using a leptospirosis case definition including both confirmed and probable cases, is 102 cases per 100,000 persons/year.

## Discussion

Our leptospirosis incidence estimate in two districts in the Kilimanjaro Region of Tanzania, among the first estimates from sub-Saharan Africa, shows a high annual incidence ranging between 75 and 102cases per 100,000 persons. This likely underestimates the total incidence of leptospirosis as it represents hospitalized cases and does not account for milder cases seeking care in outpatient settings or at home. Our leptospirosis incidence estimate is comparable to estimates of *Salmonella* Typhi incidence in Pemba, Tanzania in 2009–2010 [5]. Despite an incidence comparable to more recognized infections in this area, such as *Salmonella* Typhi, leptospirosis currently remains a neglected and underdiagnosed cause of febrile illness in Tanzania [17].

Other studies and groups such as the World Health Organization (WHO) Leptospirosis Burden Epidemiology Reference Group (LERG), which aim to assess worldwide leptospirosis incidence and burden of disease [24], have encountered scarce data from Africa on which to base assessments [6], [25], [26]. Although leptospirosis is known to occur in sub-Saharan Africa, the limited data available are based mostly on serologic surveys or outbreaks, from which incidence cannot be reliably calculated. To our knowledge, the only other published leptospirosis incidence estimate from Africa is from the Seychelles, where incidence in 1995–1996 was estimated at 101 cases per 100,000 persons [13]. This estimate is consistent with our estimate of incidence in northern Tanzania.

Our data show the highest incidence of leptospirosis in children in the age groups 0 to <5 years and 5 to <15 years, with a substantially lower incidence in those age ≥15 years. This age distribution differs from data reported elsewhere, in which adults are more commonly affected [26], [27]. The age distribution reported here could have a number of possible explanations. First, because we enrolled only hospitalized cases, it is possible that the threshold for hospital admission is lower in children than adults, which would result in an over-representation of cases among younger age groups. A lower admission threshold for children has been proposed in another study that compared leptospirosis clinical presentations between children and adults [28]Conversely, it is also possible that children were hospitalized more often than adults because they had more severe leptospirosis manifestations, but this explanation would be inconsistent with evidence from other studies [28], [29]. Additionally, it is possible that in northern Tanzania, children may have similar or greater exposure to risk factors for leptospirosis compared to adults. Rather than occupation-related exposures seen in some areas, risk could be conferred primarily through widespread environmental contamination, with exposures occurring during daily activities such as bathing in contaminated water sources or walking barefoot in mud. This could result in frequent exposures to *Leptospira* early in life. It is not known whether naturally acquired protective immunity may occur in endemic settings and what role this may play in age-related risk [30]. Further research is needed to explore the epidemiology and age-related risks for leptospirosis in sub-Saharan Africa.

The incidence estimate that we calculated represents our best effort to contribute leptospirosis incidence data for sub-Saharan Africa, where data are scarce, but we recognize a number of limitations. While the best way to estimate the incidence of febrile illnesses is by active surveillance in a well characterized population using conventional standard diagnostic methods, this approach has substantial logistic and cost barriers in resource limited settings. The multiplier method we employed has been described and applied as a means of estimating febrile illness incidence in areas where resources and infrastructure for active population-based surveillance are limited [1], [4], [5]. Multiplier methods are also well accepted by surveillance networks that attempt to approximate incidence in the face of incomplete assessments at various steps in the surveillance pyramid [31]. However, multiple assumptions are made in the derivation and application of multipliers. We assumed that those who presented to the referral hospitals under surveillance were representative of those who presented to other hospitals within the two districts. Surveillance sites are best chosen in light of findings of the health care utilization survey [2]. However, our health care utilization survey was performed after selection of our fever surveillance sites. We also assumed that there was no difference between patients who were enrolled and those who were eligible and not enrolled.

In the health care utilization survey study population, we did observe differences in age group distribution and sex compared to the general census population for the study districts, indicating that our HCUS study population was not entirely representative of the general population Although we hoped to minimize this with our selection methods, this is not entirely unexpected given the large size of the census population relative to our sample size. Additionally, multiple responses from a single household were analyzed if the household contained members in more than one designated age group. This methodology has the potential to introduce design effect given that the head of household may be likely to respond similarly to health care seeking questions for different age group members within a single household. In order to explore effects of our multiplier choices, we evaluated the effect of using more than one leptospirosis case definition and the use of different health care utilization survey questions, including a question that was answered only once for a given household. This provided a range of plausible incidence estimates, from which we selected the estimate we believed was most representative and inclusive of available data.

We also recognize that our study, conducted over 1 year in 2 districts may not be generalizable across years or to other areas in Tanzania or Africa. Leptospirosis transmission varies based on climatic conditions such as rainfall, temperature, humidity and other climatic factors [32], [33]. Our surveillance continued for a full year, thus avoiding bias from seasonal fluctuations in incidence, but incidence could be heterogeneous across years. Additionally, differences in climate, elevation and other leptospirosis risk factors such as population and livestock density, water and sanitation, and types of housing may limit generalizability across other areas in Tanzania or Africa.

### Conclusions

We calculated a high incidence of leptospirosis in 2 districts of the Kilimanjaro Region in Tanzania. Despite its high incidence, leptospirosis remains an under-recognized cause of febrile illness in Africa, resulting in a lack of resources dedicated to defining risk factors and implementing public health control measures. The high estimated incidence underscores the importance of prioritizing further research into the epidemiology of leptospirosis in Africa. There is an urgent need for rapid diagnostic tests that are sensitive and specific as well as improved surveillance in order to better assess leptospirosis case fatality and disease burden across different types of health care facilities in Africa. An approach similar to ours, using health facility based surveillance and health care utilization surveys, is a practical method that may be feasible across multiple sites with limited resources to improve leptospirosis incidence data.

## Supporting Information

Checklist S1 STROBE checklist.
(DOC)
